# Supplementary material for: Long COVID and its associated factors among COVID survivors in the community from a middle-income country—An online cross-sectional study
Source: PLoS One. 2022 Aug 30;17(8):e0273364. doi: 10.1371/journal.pone.0273364 (PMC9426885; doi:10.1371/journal.pone.0273364)
Supplement: Attachment — Submitted filename: Rebuttal to reviewers comments (PLOS One).docx [file pone.0273364.s002.docx]

| Reviewer #1: Dear EiC,  Thanks for sending me this manuscript. The survey raises an interesting point despite its local origin.  I have few recommendations to be done before further consideration to Publication: 1) The CRONBACH’S ALPHA should be calculated to test reliability between questions.  2) Why Table 4 is adjusted to sex only ? Why not to age and sex?  3) Also, comparison to same level countries like Egypt in the following paper is highly recommended to be implemented in the discussion section as: Sabry, Nirmeen et al. “Awareness of the Egyptian public about COVID-19: what we do and do not know.” Informatics for health & social care vol. 46,3 (2021): 244-255. doi:10.1080/17538157.2021.1883029  4) Also, I recommend to add this citation in the discussion section as potential solution for the coming infectious outbreaks: the Pharmacists as Healthcare providers in their Community Pharmacies can act as an important and early screening and testing point for COVID-19 infection such as in this mini-review: <https://doi.org/10.1177/08971900211036093> | Thank you for your suggestion. The answers to the questions asked in our questionnaire are yes / no. However, Cronbach's alpha is used to test if multiple-question Likert scale surveys are reliable. We did not include any Likert scale questions in the current research.  Therefore, Cronbach’s alpha is not imputed.  Sorry for the typo error. The analysis was adjusted for age as well. Have added in the footnote.  Thank you for the suggestion. However, our paper focus on symptoms of Long COVID, not about COVID-19 at the acute phase.  Thank you for the suggestion. Our discussion focus on Long COVID, not COVID-19 infection at the acute phase. |
| --- | --- |
| Reviewer #2: Dear author, I think the tables can be improved especially table 4. There's repetitive us of the word 'reference; within the table.  Overall, the paper needs some minor editing especially on punctuation. For example Long COVID, Central region, Intensive Care Unit. There's no need for capital letters. | The “reference” refers to the group used as reference for each analysis. There are two sets of analyses, bivariate and multivariable analyses. The bivariate analysis provided the crude Odds Ratio and the multivariable analysis provided the adjusted Odds Ratio.  Long COVID is a well-accepted way of presentation by other researchers. Central region is edited as central region, while Intensive Care Unit will remain as it is. |
